# Bioengineering the Vascularized Endocrine Pancreas: A Fine-Tuned Interplay Between Vascularization, Extracellular-Matrix-Based Scaffold Architecture, and Insulin-Producing Cells

**DOI:** 10.3389/ti.2022.10555

**Published:** 2022-08-25

**Authors:** Cataldo Pignatelli, Francesco Campo, Alessia Neroni, Lorenzo Piemonti, Antonio Citro

**Affiliations:** ^1^ San Raffaele Diabetes Research Institute, IRCCS San Raffaele Scientific Institute, Milan, Italy; ^2^ Università Vita-Salute San Raffaele, Milan, Italy

**Keywords:** type 1 diabetes, beta cell replacement, bioengineering, vascularized endocrine pancreas, 3D- bioprinting, extracellular matrix, biomaterials, alternative endocrine sources

## Abstract

Intrahepatic islet transplantation is a promising β-cell replacement strategy for the treatment of type 1 diabetes. Instant blood-mediated inflammatory reactions, acute inflammatory storm, and graft revascularization delay limit islet engraftment in the peri-transplant phase, hampering the success rate of the procedure. Growing evidence has demonstrated that islet engraftment efficiency may take advantage of several bioengineering approaches aimed to recreate both vascular and endocrine compartments either *ex vivo* or *in vivo*. To this end, endocrine pancreas bioengineering is an emerging field in β-cell replacement, which might provide endocrine cells with all the building blocks (vascularization, ECM composition, or micro/macro-architecture) useful for their successful engraftment and function *in vivo*. Studies on reshaping either the endocrine cellular composition or the islet microenvironment have been largely performed, focusing on a single building block element, without, however, grasping that their synergistic effect is indispensable for correct endocrine function. Herein, the review focuses on the *minimum* building blocks that an ideal vascularized endocrine scaffold should have to resemble the endocrine niche architecture, composition, and function to foster functional connections between the vascular and endocrine compartments. Additionally, this review highlights the possibility of designing bioengineered scaffolds integrating alternative endocrine sources to overcome donor organ shortages and the possibility of combining novel immune-preserving strategies for long-term graft function.

## Introduction

Type 1 diabetes (T1D) is characterized by autoimmune-driven destruction of insulin-producing β-cells, which leads to altered control of glucose homeostasis and induction of hyperglycemia. The first line treatment is the exogenous insulin administration *via* multiple daily injection (1–4). An alternative strategy to insulin injection is to replace the endocrine mass by transplanting allogeneic pancreas or pancreatic islets in T1D patients experiencing insulin-dependent hypoglycemia unawareness, severe hypoglycemia, and unstable glycemia (5–9). To date, pancreas transplantation is more frequently used in clinical practice than islet transplantation, although it has more important surgical procedures. Indeed, islet transplantation is an easy and poorly invasive procedure that avoids post-surgery burdensome effects on patients (10). Islet transplantation has a high success rate in alleviating hypoglycemic events and improving the quality of life of patients. However, only a small percentage of recipients acquire insulin independence after intrahepatic islet transplantation. A gradual loss of both graft function and insulin independence was observed within 5 years of islet implantation (7,8,11). Despite the short-term function, the results derived from the recipients demonstrated that reestablishing endocrine pancreatic function has the potential to restore fine endogenous control over glucose homeostasis, which cannot be precisely mimicked by closed-loop artificial pancreas devices (12,13).

The inability to achieve long-term function of the intrahepatic islet graft must be sought 1) in the inflammatory processes in the peri-transplantation phase, leading to early graft loss, 2) in the missed prompt vascularization, and 3) in allo-immune reaction and autoimmune recurrence (14–16). In particular, instant blood-mediated inflammatory reaction (IBMIR) leads to a loss of approximately 50%–70% of the total infused islet mass within the first few hours to days after transplantation. Additionally, at the hepatic site, tissue reperfusion-related damage and thrombotic events further increase the inflammatory state, leading to poor engraftment efficiency (17,18). Furthermore, the delay in functional graft vascularization dangerously exposes islets to hypoxic stress and lack of nutrients for at least 2 weeks after transplantation, causing islet cell death and apoptosis (19). To balance this intrinsic procedure limitation, a high number of islets are infused, with at least 10000 islets equivalents (IEQ)/kg body weight generally obtained from two or three donor pancreata, increasing the overall organ demand (20,21). On the other hand, to avoid immunological reactions against the graft in the post-transplant phase, life-long immunosuppressant administration is provided, which in turn can provoke kidney failure and increase cancer risk and infection (15). In recent years, alternative transplantation sites have been proposed to increase the success rate of allogeneic islet transplantation; however, to date, no one has shown superior outcomes compared to the intrahepatic site (6,7,11).

In this scenario, to overcome the current limitation and improve outcomes, several points need to be achieved: 1) the identification of an alternative site with a microenvironment architecture that may improve endocrine function; 2) fostering prompt vascularization, able to ensure an adequate exchange of oxygen, nutrients, and hormones to support endocrine pancreatic cells in effectively sensing blood glucose changes; 3) the identification of a method to mitigate the innate immune reaction to avoid early graft loss; 4) the definition of alternative strategies granting long-term graft immune protection; and 5) the identification of a renewable source of insulin-secreting cells to widen the treatment to a larger cohort of T1D patients (22). To achieve these goals, tissue engineering (TE) approaches can provide new insights, especially in increasing vascularization at transplantation sites through biomaterial-based strategies. Indeed, the intention of the last years has been to recreate a vascularized site to accommodate endocrine cells in order to accelerate graft revascularization and shorten the hypoxic phase. Although these approaches have been largely investigated in clinical trials, research in this field is moving towards the design of systems resembling the endocrine native niche, especially considering its organization, in terms of supporting cell type and microarchitecture. Introducing these two components into bioengineered systems may support structural and functional integration between the endocrine and vascular compartments, which is fundamental for recreating the physiological microenvironment of the endocrine niche and improving the biocompatibility of the graft with the host tissue (23).

TE technologies may also give the chance to recreate endocrine pancreas using alternative endocrine sources, such as pluripotent stem cells (PSCs) or xenogeneic source appropriately modified, favoring the exploration of their function and the feasibility of the approach in clinical practice (11,24). Finally, the flexibility of TE technologies might help overcome the systemic administration of immunosuppressive drugs by combining novel immunosuppressive strategies to locally achieve an immune-privileged transplantation site (15,23,25,26). To overcome the limitations of classical β-cell replacement, bioengineered endocrine pancreas systems need to be inspired by the native niche. Therefore, we will first define the native endocrine niche architecture and functional components and subsequently address TE strategies for tuning and reshaping.

## The Endocrine Niche

**FIGURE 1:**
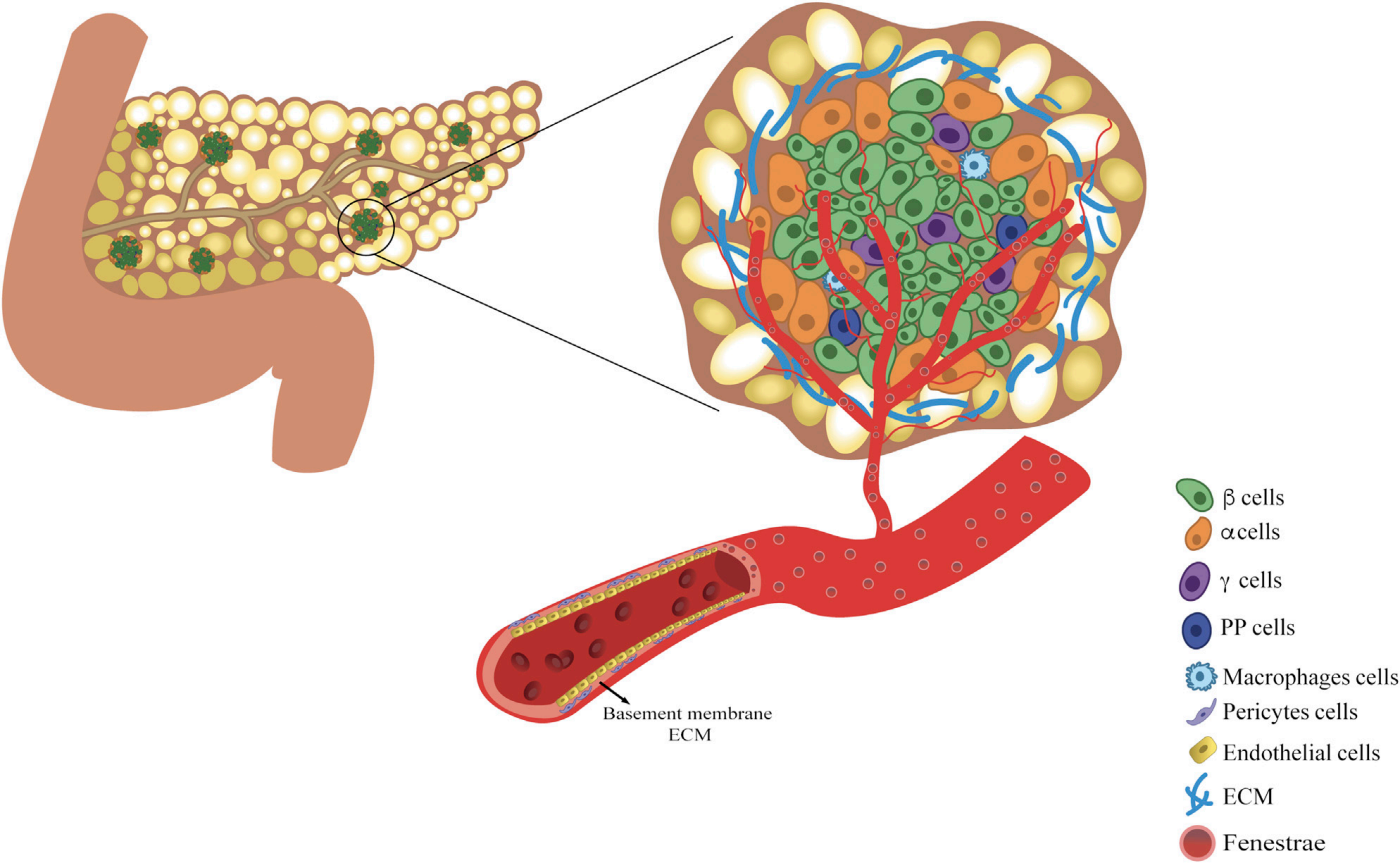
The vascularized endocrine niche within the pancreatic tissue. Pancreatic endocrine niche is enclosed within the pancreatic exocrine tissue and it is constituted by three main components: the extracellular matrix, islet of Langerhans and fenestrated vascular network. Islet is mainly composed of insulin-secreting β-cells, glucagon-secreting α-cells, somatostatin-secreting δ-cells, pancreatic polypeptide-secreting PP-cells, and macrophages. The microvasculature within the endocrine cell cluster is fundamental both for sustaining the endocrine cells viability and for accomplishing for their function.

Human pancreas is a unique and complex organ that contains both exocrine and endocrine tissues. The exocrine part accounts for 98% of the organ parenchyma and secretes pancreatic juice into the duodenum for correct digestion and assimilation of nutrients (27). The endocrine compartment represents the remaining 2%. The endocrine side is organized into independent cluster units (27) scattered throughout the exocrine parenchyma, best known as the islet of Langerhans (27,28). They are embedded within a capsule consisting of an extracellular matrix (ECM) and fibroblasts, in which endocrine cells are non randomly aggregated. Islets are independently fed by a dense network of highly fenestrated capillaries, which allows each endocrine cell to be in close contact with the blood (28). Specific organization of the ECM, cells, and microvasculature consitutes the endocrine niche (Figure 1). Owing to the evaluation of the endocrine niche, it was possible to identify the fundamental features useful for bioengineering endocrine pancreatic tissues. Thus, the role of each component will be briefly reviewed, identifying it as an essential part of the niche microenvironment that synergistically supports endocrine functions.

### Cell Roommates of the Endocrine Pancreatic Niche

Among endocrine pancreatic cell types, β-cells are the most abundant, accounting for 60%–75% of islet cells, constituting the sole source of cells capable of secreting insulin and amylin. α-cells are the second most abundant cells (20%–30%), secreting glucagon as an insulin counter-regulatory hormone. Other endocrine cells are δ, ε, and pancreatic polypeptide cells (PP), which release somatostatin, ghrelin, and PP hormones, respectively (28). According to the work of Bonner-Weir et al., differences in cell composition based on islet dimensions have been observed: large islets have a lower content of β-cells compared to medium-sized islets (∼60% vs. ∼75%) (29). Additionally, most medium- and small-sized islets have a non-random organization. They present a layer of β-cells between the two layers of α-cells. Large islets display a more random organization owing to their low β-cell percentage (29,30).

All endocrine cells work together to establish a complex paracrine network that ensures proper control of blood glucose levels (31,32). In addition, interactions between endocrine cells and other roommate microenvironments, such as vascular and innate immune cells, are essential for the correct development and function of the endocrine network (33).

Vascular cells, such as endothelial cells (EC) and pericytes, generally constitute the cellular part of the *tunica intima* of vessels, while the structural part is the basement membrane (BM), which is constituted by a specific ECM. In the endocrine pancreatic niche, ECs form a fenestrated endothelium, guaranteeing high permeability (ten times more fenestrae compared to exocrine vessels) and a greater capacity for nutrients, hormones, oxygen, and metabolic waste exchange (34,35). ECs can directly affect β-cell function by upregulating insulin secretion and promoting β-cell survival via the secretion of soluble factors such as hepatocyte growth factor (HGF), vascular endothelial growth factor (VEGF)-A (36) and ECM proteins (37).

Although the contribution of EC to islet endocrine function has been well described in the literature, the role of PC has recently been emphasized (35). Pericytes are abluminal mural cells embedded in the BM-ECM of blood vessels and play a key role in regulating endocrine niche homeostasis and function (35,37). Indeed, Landsman et al., in a series of ablation experiments, reported the role of pericytes in β-cell expansion during the neonatal stage and in the maintenance of β-cell maturation and function in adulthood (38–40), regulating the production and deposition of islet ECM components and promoting the expression of β-cell genes including Ins1, Mafa, and Glut2 (41). Additionally, pericytes are directly involved in β-cell function through nerve growth factor (NGF) pathways, activating the release of insulin granules in the presence of high glucose levels (42).

Along with vascular cells, macrophages reside in the endocrine niche (43) and participate in maintaining tissue homeostasis and/or dysfunction (44). Studies on mice have revealed that resident macrophages are present in the prenatal stage, constituting a pool of tissue-resident macrophages maintained by local proliferation (45). Two different subsets have been identified by immune profiling: F4/80^lo^CD11c^+^ macrophages present within the islet structure and F4/80^hi^CD11c^–^ macrophages largely residing in the peripheral islet area (46). Both subsets are in close contact with vasculature and endocrine cells and act as sensors; they sense and respond to cues modulating their activation state and release proliferative factors, such as tumor necrosis factor-alpha, interleukin (IL)-6, IL-10 (47), insulin growth factor-1 (48) and transforming growth factor-beta, which have been demonstrated to sustain β-cell mass viability (43). Saunder et al. confirmed the synergistic network between roommates, demonstrating a coordinated interaction between EC and resident macrophages in promoting β-cell regeneration. They also highlighted the key role of ECM-mediated signaling and remodeling (49). Taken together, each roommate presents peculiar features and tasks in efficiently supporting the endocrine compartment and its function, which makes it an optimal candidate to consider and integrate in bioengineering a platform.

### Islet ECM Composition and Architecture

Physiologically, the ECM provides mechanical and physical support to cells and affects cell migration, proliferation, and differentiation (50,51). It is a three-dimensional network composed of fibrous-forming proteins, such as collagens, laminins, glycoproteins, elastin, and glycosaminoglycans (51,52). In the pancreas, BM is predominant: it surrounds the acinar cells of the exocrine pancreas, duct vessels, and pancreatic islets (53). More specifically, pancreatic islets are embedded in ECM-based structures with a specific and balanced protein composition, hierarchical organization, and determined architectural features, which are strictly related to the correct endocrine function (54–57). Islet ECM can be subdivided into an external and incomplete peripheral capsule, the peri-islet ECM, and an internal ECM, the inner matrix (IM) (58). They are secreted from different cell types; the former is secreted by exocrine cells (59), while the latter is the vascular BM secreted by vascular cells (35). As endocrine cells are not able to secrete ECM proteins, VEGF-A secretion from β-cells recruits EC to induce ECM deposition and maintain homeostasis (57,60,61). The islet inner ECM of humans has unique features: endocrine cells and islet capillaries are separated by double leaflets of vascular BM (30,62,63). The ECM composition of the endocrine niche varies during human development, as different protein isoforms are expressed from early tissue precursors to mature human pancreas (64). Although there is no consensus on islet ECM composition, the predominant proteins are collagen type IV, laminin, and fibronectin with various prevalence (65). Collagen type IV contributes to BM formation (66) and favors the maintenance of the capsule architecture. Collagen IV binds *α*_*1*_ *β*_*1*_ integrin expressed on β-cells, inducing essential signals for islet development, enabling migration of fetal β-cells, and forming normal islet architecture (67). It also enhances islet adhesion, proliferation, and insulin secretion (68). Laminin exists in several isoforms in the islet niche, and although the cell responsible for producing each isoform is still unclear, some studies have defined temporal and spatial expression. Laminin-111 is the primary isoform expressed during pancreatic development that promotes β-cell differentiation (69). During islet maturation, laminin-111 is completely replaced by the laminin-511, -521, -411, and -421 isoforms. In mature endocrine tissue, the BM leaflet towards the endocrine cells displays laminin-511, while the leaflet of the vascular lumen also laminin-411, -421 and 521, besides laminin-511 (62). Laminins bind to different integrin and non-integrin receptors on β-cells, such as *β*1 integrin, α*V* integrin, and dystroglycan (58). As a result, the interaction in β-cells activates several signaling cascades aimed at enhancing insulin secretion, inducing the expression of islet-specific transcription factors such as PDX1, Ins1, Ins2, glucagon, somatostatin, and GLUT-2 (70) and promotes β-cell survival and proliferation (59). Fibronectin is a multifunctional component of ECM that facilitates cell adhesion. It interacts with the arginine-glycine-aspartic acid (RGD) receptor to improve islet function, β-cell proliferation, and glucose-stimulated insulin secretion. The interaction with fibronectin-RGD induces the expression of differentiation markers for endocrine tissues, such as PDX1 and Ins2 (70) and improves islet cell survival, boosting the expression of anti-apoptotic protein BCL-2 (71). This evidence supports the idea that ECM components play beneficial roles towards endocrine pancreatic cells. In conclusion, the ECM was originally thought to exist to solely provide structural support to cells and it is now recognized as a reservoir of information contributing to tissue homeostasis and function (51).

### Vasculature

Although the endocrine compartment represents 2% of the pancreatic mass, it receives about 15%–20% of the pancreatic blood flow (9). Islets have a highly specialized network of arterioles, capillaries, and venules, known as the microvasculature. Owing to the high density and fenestration of capillaries, endocrine cells are bathed by blood, allowing a rapid exchange of nutrients and hormones, which is essential to correctly control blood glucose levels. Depending on their dimensions, each islet is in contact with 1-5 arterioles, which are divided into capillaries enveloping the islet and generating a structure similar to a renal glomeruli (72–75). If small islets have their own microvasculature organization, large islets have been proposed to be organized in small endocrine subunits, independently fed by proper but similar microvasculature (30). Several hypotheses have been proposed to model islet blood flow and its correlation with endocrine function, given its importance in the rapid sensing of blood glucose fluctuations and the corresponding counterbalancing hormone outflow (76–78). Three models of islet flow, which are not mutually exclusive, have been proposed and supported by studies on mice. In the first model, peripheral-to-center blood flows from the exterior to the interior of the islet. According to this model, islets are composed of a β-cell core surrounded by an α-cell layer, which is the first layer exposed to blood flow. Thus, α-cell secretagogues might directly influence the function of the β-cell core (76). In the second model, center-to-periphery, the blood flow reaches the β-cell core and then flows to the periphery where the α-cells are located. Products from β-cells can directly influence α-cells (77). In the third model, the pole-to-pole arterioles simultaneously contact all cell types in different islet regions (78). However, it is worth underlining that the architecture of the islet varies across species and β-cells are not always confined to a central core, as in humans, and in some species, a totally opposite islet organization can be found (79).

## Tuning the Endocrine Niche

In the field of β-cell replacement, recreating the endocrine niche *ex vivo* might be advantageous, as it could overcome the current limitations of clinical treatments in T1D. Deep investigations of the physiology of the native endocrine pancreatic niche have helped to understand the principal features useful for bioengineering vascularized endocrine pancreas. In addition, other evidence has been derived from the comprehension of the mechanisms involved in the failure of engraftment upon transplantation at different sites (7,11).

### Vascularization and Oxygenation of Transplantation Site

**FIGURE 2:**
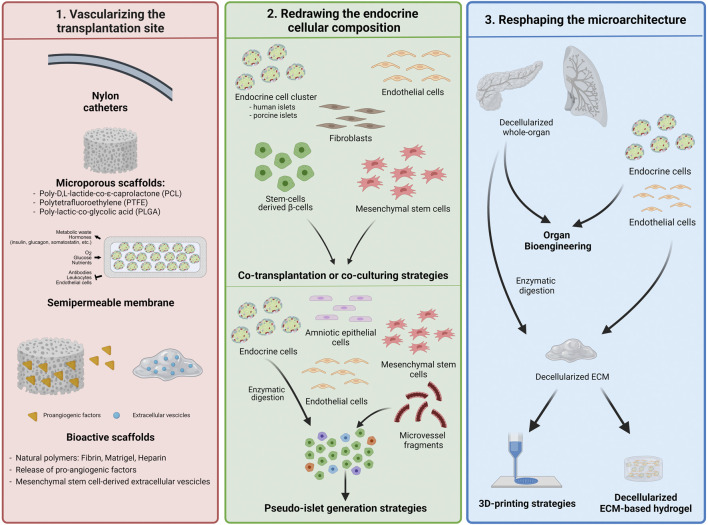
Bioengineering the vascularized endocrine pancreas—building blocks assembly. Strategies mostly used for recreating the endocrine niche in order to improve the endocrine cells viability, their engraftment and function. All of them are aimed to accelerate the vasculature-building block to shorten the hypoxic with different approaches.

The endocrine niche is not only deeply vascularized, but the vascular architecture is also based on hierarchical vessel distribution, which rules oxygen diffusion, nutrient distribution, and hormone secretion, affecting the physiological endocrine function (6,9). These features can explain the sensitivity of endocrine pancreatic cells to hypoxic environments and a lack of nutrients (80). After the isolation process and in the early transplantation phases, islets are completely deprived of vascularization and the correlated oxygen and nutrient supply until engraftment within the host tissue, which occurs upon the re-establishment of functional vascularization in 1–2 weeks (19). Based on this evidence, β-cell replacement strategies are focused on finding vascularized sites, evaluating alternative transplantation sites compared to the liver, or preconditioning strategies that increase vessel density at the implantation site. Owing to the failure to find alternative transplantation sites, the preconditioning strategy has gained ground, especially by exploiting biocompatible materials (7,11,81,82). In particular, engineering a transplantation site to increase vascularization is thought to be suitable for ameliorating the engraftment and function of endocrine pancreatic grafts (83) (Figure 2). Implantation of nylon catheters or cylindrical stainless-steel mesh tubing in rodents helped to create vascularized pouches in 1 month, exploiting the foreign body response (FBR) without inducing scar formation in different tissues. After removal, syngeneic islets or human islets were easily implanted, showing the ability to reverse diabetes in the respective appropriate rodent models (84), while islets infused in the not-preconditioned pouch were not able to restore normoglycemia (85–89). Similarly, poly-D,L-lactide-co-ε-caprolactone (PLCL)-based scaffolds were used to pre-vascularize the subcutaneous space after 1 month. The islets were positioned in channel structures, which were closed using polyethylene tubing. The system restored normoglycemia in recipient mice with a similar trend as that in kidney capsule recipient mice (90). Exploiting the capability of materials to induce vascularization of the implantation site through FBR, clinical studies have been performed on vascularizing systems. Another study demonstrated the possibility of creating a subcutaneous cavity using a non-degradable Silon monofilament mesh in a murine immune-deficient diabetic model for islet implantation. This study showed that rat islets, in combination with additional supporting cells, were able to engraft and restore normoglycemia for up to 4 months. However, additional investigations are required to further validate this promising approach (91).

However, devices investigated in clinical trials do not consider the pre-vascularizing phase, such that the second surgery for islet positioning is avoided. ViaCyte Encaptra (NCT02239354, NCT03163511) and Sernova Cell Pouch (NCT03513939) are devices that encapsulate insulin-producing cells that induce vascularization of the subcutaneous site upon implantation. Particularly, ViaCyte (VC-01), which is based on Theracyte technology, was composed of a semipermeable membrane to allow oxygen and nutrient exchange but at the same time to isolate transplanted cells from the recipient immune system. The trial was suspended because of poor survival and engraftment of the transplanted cells due to FBR, which clogged the membrane, preventing nutrient exchange and vascularization (92). Additionally, the presence of an immune-isolating membrane hinders the recipients’ capillaries formation, which, therefore, are not able to recreate the native islet perfusion and connection. In 2017, ViaCyte started a second trial using a modified encapsulation device (VC-02). The system did not provide complete immune isolation but allowed vascular permeation through the presence of dedicated pores on the device surface. The upgraded design of the device showed a great improvement in the final outcome by the detection of secreted C-peptide in T1D patients within the first year (63%) (93,94). The Sernova cell pouch was similarly aimed to recreate a suitable subcutaneous microenvironment for islet implantation, and a clinical study is still ongoing with patients in an immunosuppressive regimen (7). However, further studies are needed to evaluate the long-term functions of these devices (Figure 2).

The aforementioned systems use materials with inert features and are unable to actively interact with the surrounding tissue because they are neither provided by recognition sites for cells nor by biological stimuli. The introduction of bioactive molecules and/or bioactive materials enables the finalization of several mechanisms for device vascularization and colonization of host cells (25). Natural polymers have been widely investigated to enhance the vascularization of transplantation sites (Figure 2). Kuppan et al. used a poly lactic-co-glycolic acid (PLGA)-based fibrous scaffold modified with gelatin polymer, which was implanted in the abdominal subcutaneous space for 4 weeks to induce site vascularization. Implantation of xenogeneic islets has reversed the hyperglycemia within 20–25 days, similarly to mice with islets implanted at the kidney capsule site (95). Fibrin has been widely used as the Food and Drug Administration (FDA) approves its clinical use (25). It is a fibrous protein derived by the self-assembly of fibrinogen molecules upon cleavage by thrombin during the coagulation process, and helps the revascularization during wound healing as it presents RGD domains, which induce cell survival and migration (25,96,97). Fibrin hydrogel was previously used to encapsulate islets, which were then transplanted into the omental pouch of diabetic rats or diabetic *Cynomolgus* monkeys. The hydrogel reversed hyperglycemia, gradually reducing the exogenous insulin need, and efficiently supported optimal graft revascularization (98). The promising results in preclinical models led to an ongoing clinical study of the BioHub platform (NCT02213003) (98,99). However, the results at 1 year follow up after transplantation showed decreased graft function. According to the authors, the recipients lost insulin independence over time due to a switch in immunosuppressive regimen from tacrolimus to sirolimus administration (99,100). Fibrin is also involved in IBMIR and prudence is therefore required to avoid the presence of complement proteins in fibrin batches.

Among the commercially available native biomaterials, murine sarcoma-derived hydrogel-Matrigel™ and heparin were also used. They were positioned in a silicone cylinder tubing at the mouse groin, which was closed at the distal end with inguinal fat and completely sealed with bone wax. The study showed the ability of the chamber to obtain a microvascularized network after 28 days and to sustain the engraftment and function of syngeneic islets after 10–14 days (101,102). However, the use of Matrigel in clinical procedures has some shortcomings related to its poorly defined chemical composition (25).

Vascularization strategies using synthetic or natural materials have shown the ability to create a microenvironment that is more comfortable for islet accommodation at transplantation sites, as highlighted by previous reports. For preconditioning strategies, there is the disadvantage of a second surgery for positioning insulin-producing cells. Additionally, the triggering of FBR is due to the recruitment of neutrophils, macrophages, and mast cells, which normally react against a material-based implant, generating a fibrotic capsule around it, isolating the endocrine pancreatic graft, and finally inducing the formation of an unorganized vessel network that is not properly functional in a fully vascularized graft (103). However, these strategies still present positive and relevant aspects, considering the delay of technological improvement, which hinders the development of new strategies clinically relevant for β-cell replacement.

The ViaCyte experience has highlighted the crucial role of prompt and complete vascularization upon implantation. Therefore, to provide a scaffold with optimal proangiogenic capability, several studies have evaluated the addition of growth factors such as VEGF, angiopoietin-1 and 2 (Ang1 and Ang2), platelet-derived growth factor (PDGF)-BB, and fibroblast growth factor (FGF)-2 (104–109). The controlled and sustained release of single or multiple factors from the scaffolds has been demonstrated to induce both angiogenesis and the formation of mature vessels with respect to a random mix of growth factors within the hydrogel or direct injection at the implantation site (110–112) (Figure 2). In fact, the tailored release of growth factors allows the creation of gradients that attract recipient ECs towards the implantation site (113). In previous studies, VEGF has been released in a sustained manner through chemical binding to polymeric scaffolds or by exploiting the growth factor-binding ability of heparin. Sustained release of VEGF over time improved islet engraftment because of higher cell penetration, which allowed the formation of new capillaries than islets embedded into free VEGF-polymeric scaffolds (104,113–115). Similarly, multiple or sequential release of different proangiogenic factors from the implanted scaffold might be another approach for increasing new vessel formation at the graft site. The release of VEGF followed by PDGF or FGF-2 has been shown to increase the maturation of vessel networks compared to VEGF alone. FGF-2/VEGF co-release has been proposed to mimic physiological secretion in the vascularization process during wound healing. Polylactic acid (PLA) fibrous scaffolds modified with heparin-binding amphiphilic peptides could store and slowly release VEGF and FGF-2. Recipient mice receiving the modified fibrous scaffolds with islets reversed the hyperglycemia faster than control mice receiving the bare fibrous scaffold, thus suggesting the ability of the modified scaffold to sustain islet engraftment and function (116). To this end, platelet-rich plasma (PRP) is suitable for multiple factors release for revascularization. It has a growth factor composition in a ratio that is useful for efficient vascularization. Indeed, it is used for chronic wound healing treatment (117) and cell transplantation as a coating of a PLA-based chamber to induce vascularization in the subcutaneous space of mice (118).

Recently, with the idea to promote angiogenesis in a biomimetic manner, several studies have focused on introducing multiple proangiogenic stimuli mimicking the multi-combinatorial aspects of physiological processes (109,119–121). Knowing that islets physiologically are able to secrete factors for recruiting ECs, Staels et al. considered the possibility of enhancing the capability of transfecting islets with an mRNA encoding VEGF, showing that vessel formation was increased (119). Xing et al. proposed the use of mesenchymal stem cell (MSCs)-derived extracellular vesicle (EVs) chemo-selectively immobilized onto a collagen-based scaffold. This strategy induced higher host cell infiltration and improved angiogenesis, including vascular ingrowth and macrophage recruitment, compared to scaffolds without immobilized EVs (121). Similarly, Najjar et al. proposed the use of a fibrin-based gel complexed with a recombinant human fibronectin fragment containing integrin and binding domains for VEGF and PDGF. Thus, although the gel had minimal doses of VEGF and PDGF-BB and was loaded with a marginal mass of syngeneic islets, the interaction of both VEGF and PGDF receptors with integrin *α*_*5*_ *β*_*1*_ through fibronectin domains enhanced revascularization. The induced vascularization showed a higher ability to reverse hyperglycemic conditions compared to the non-complexed and unloaded hydrogels upon implantation in the epididymal fat pad in preclinical models of diabetes. This was positively correlated with the prompt revascularization induced by the fine assembly of the gel with encapsulated VEGF and PGDF (109,120).

Overall, these results highlight that releasing multiple factors in a biomimetic manner might enhance the recruitment of proangiogenic cells, accelerating vessel formation. However, it is not enough to recapitulate either the physiological mechanisms of angiogenesis or the impact of ECM components on vascular regeneration through cell-cell and cell-matrix interactions. Therefore, structural support is required to achieve more efficient and physiological vascularization (25) (Figure 2).

A different strategy to support β-cell viability and function upon implantation reduces the physiological latency of the vascularization process using oxygen-producing devices. A clinical study evaluated the β-Air bio-artificial pancreas, which had a daily refillable oxygen chamber between two layers of alginate encapsulating the islet to maintain an adequate oxygen supply (NCT02064309) (98,112,122). β-Air improved cell viability and supported graft function, which were detected for 10 months without immunosuppression. However, this strategy still cannot ensure adequate glucose sensing and insulin release kinetics in the islets (122). Another study designed an encapsulation system that generates oxygen starting from metabolic waste products such as carbon dioxide through an inverse breathing chemical reaction. The device uses the gas-solid reaction of carbon dioxide with lithium peroxide to produce oxygen, whose pressure remains constant. However, there are concerns related to lithium peroxide toxicity and the finite oxygen supply of the device (123).

Supporting encapsulated islets through oxygen-dispensing techniques in the post-transplant phase may be useful to support their viability and long-term function, without the need to induce vascularization. However, endocrine function, especially glucose sensing, can be hindered by the lack of vascularization, which is fundamental for an efficient endocrine graft function (122).

### Redrawing the Endocrine Pancreatic Cellular Composition

Alternative approaches in the field of β-cell replacement aim to combine additional cell types with endocrine cells to foster functional vascularization and engraftment of endocrine cells in a physiological and biomimetic fashion. MSCs, EC, and fibroblasts have been used *ex vivo* and *in vivo* to reshape endocrine cell cluster composition (25,124). EC are directly involved in reconstructing the vessel as they compose the endothelial barrier and sustain the mechanism through paracrine signals, whereas MSCs and fibroblasts are known to participate in the vascularization process by supporting the EC (125–127). Additionally, MSCs enhance angiogenesis by remodeling the ECM, secreting VEGF, Ang1 and 2 and stabilizing vasculature (128) (Figure 2).

Co-transplantation of porcine islets with MSCs in diabetic mice or primates has been shown to support vascularization and normoglycemic restoration (25). Following the same strategy, other reports showed that the combination of human or rodent islets with MSCs and/or fibroblasts loaded in a collagen-fibrin hydrogel implanted in recipient diabetic mice demonstrated the positive impact of accessory cells to promote higher vascularization, earlier graft function, and better control on glucose homeostasis compared to islets alone (129–131). Additional studies have characterized the MSC subtypes and their different roles in supporting cells in the β-cell replacement approach. Forbes et al. showed that human islets co-transplanted under the kidney with MSC derived from the perivascular tissue capsule had better glycemic control than human islets implanted alone and restored normoglycemia conditions within 5 days. Comparing this work with other reports in which MSC derived from bone marrow or adipose tissues were used, perivascular MSC seemed to be more effective in rapidly restoring normoglycemia (132–137).

Human umbilical vein endothelial cells (HUVEC) were widely used as an EC model to evaluate their impact on endocrine graft revascularization. Collagen type I hydrogels embedded with rat islets and HUVEC have been shown to restore normoglycemia within 8 days, displaying a higher presence of CD31^+^ cells and proangiogenic CD206^+^/MHCII^−^ (M2-like) macrophages after 7 and 14 days compared to non-encapsulated islets (138). In addition, 24h-self-aggregation of human or mouse islets with HUVEC and human MSCs promoted both good endocrine pancreatic graft function and a massive improvement in post-transplant engraftment, suggesting the beneficial activity of the supporting cells. The authors also highlighted the role of MSCs and HUVEC in producing ECM, in particular laminin and collagen IV of the BM, which was highly observed along the EC within endocrine tissues (134). Blood outgrowth endothelial cells (BOEC) have also been used as EC model to ameliorate graft vascularization. They showed their ability to reduce β-cell death and induce good vascularization of the graft, producing metabolic benefits in diabetic immunodeficient murine models (139). Other studies used ECs or MSCs to coat islet preparations, and independent of the methods used, coated islets showed better engraftment due to enhanced vascularization. Finally, other authors have proposed specifically coating islets with proangiogenic cells rather than co-culture or co-transplantation (135–137) (Figure 2).

More recent strategies have been developed to reshape endocrine cell composition. Starting from the possibility of creating pseudo-islets by re-aggregating enzymatically digested islet cells into homogenous cell clusters, several groups have suggested combining islet cells with other cell types to enhance the pseudo-islet endocrine function (140–144). Digested rat islets in single cells were reassembled in new type of endocrine-like cluster co-aggregating HUVEC and human amniotic epithelial cells (hAEC), obtaining heterotypic spheroids with homogeneous size. While HUVEC were added to sustain the vascularization of the cellular clusters, hAEC, known as cells expressing a pluripotent and immune-modulating repertoire (140–142), were introduced to shield endocrine cells and modulate the response of the host immune system. The assembled spheroids demonstrated an enhanced *in vitro* function and, upon implantation into the epididymal fat pad of a diabetic immunocompromised murine model, faster engraftment and vascularization when compared to undigested rat islets (142). Similarly, a previous study evaluated the impact of spatial aggregation of the human β-cell line EndoC-bH3 and EC. Heterotypic pseudo-islets composed of a core of islet-derived cells surrounded by an outer layer of EC showed increased insulin secretion and, therefore, β-cell functionality, emphasizing that the spatial distribution and cell-cell interactions are features to be considered to reconstitute the organization of the pancreatic islets (143). Digested islet cells along with EC and MSC embedded in collagen type I-based hydrogel rods, further coated by other EC, were able to restore normoglycemia within 2 weeks after subcutaneous implantation in streptozotocin-induced diabetic mice, which also showed good control of glucose metabolism (144) (Figure 2).

The introduction of supporting cells into the re-shaped endocrine pancreatic constructs has been shown to increase vascularization by their direct involvement in vessel formation or by recruiting host cellular counterparts. However, their random addition to the system imperils the rapid formation of organized vessels, retarding the anastomosis with host vessels and hampering the gain of graft function. This provokes a lag in the integration of the endocrine graft in the host tissue, dangerously exposing insulin-producing cells to hypoxic stress. To further shorten engraftment time, Nalbach et al. fused murine islet-derived cells to murine epididymal fat pad micro-vessel fragments, which consist of EC-lined lumen covered by stabilizing α-smooth muscle actin (αSMA)-positive cells with preserved micro-vessel structures. The resulting organoids displayed reduced hypoxic stress, increased insulin secretion *in vitro* and faster hyperglycemia-reversing ability due to rapid revascularization compared to non-pre-vascularized organoids and fresh islets (145). These results highlight the importance of preformed structures to obtain a pro-vascularizing architecture within insulin-producing elements, which have been shown to increase oxygen penetration within the 3D organoid structure and accelerate revascularization *in vivo*.

### The Role of ECM and Microarchitecture

Pancreatic islet isolation is associated with peri- and intra-islet ECM destruction (55,56,146). Even if intra-islet ECs are still present in the first days after isolation, they undergo gradual death, compromising their islet-ECM replacement ability (56). Upon isolation, laminin significantly decreases after 24 h in *ex vivo* culture, while the remaining collagen IV remain during the culturing phase (14,56,57,147). Loss of ECM leads to β-cell cytotoxicity, apoptosis, and reduced insulin production (56,147–149). Therefore, the use of ECM-based polymers to bioengineer endocrine pancreas is another necessary aspect to consider. ECM proteins, alone or in combination with synthetic materials, have been used to fabricate scaffolds for β-cell replacement (54,56,57,150–155). However, the microenvironment of the pancreatic endocrine side is characterized by a specific ECM design with a fine balanced protein composition; therefore, a simple mixture of ECM-derived polymers may not be sufficient to reproduce the complexity of the mechanobiology involved (156). This specific “intrinsic design” is not only structurally strategic for cell-to-cell interactions but also functionally relevant for tuning endocrine function. Several studies decellularized whole organs through detergent perfusion, preserving the entire ECM organization as well as the microarchitecture with the aim of taking advantage of the native organ ECM structure and composition (Figure 2). Decellularized lungs (157–159), kidney (160), spleen (161), liver (162–164) and pancreas (165–172) were recellularized by exploiting the pre-existing macro- and micro-architecture of native organs to recapitulate the complexity of the native endocrine microenvironment (173,174). As expected, decellularized scaffolds preserved native ECM composition, confirming that decellularization did not alter the chemical and physical properties of the native organ (170). However, glycosaminoglycan loss can occur, depending on the decellularization protocol, leading to an increase in both the stiffness and Young’s modulus of the decellularized scaffolds (175). Seeding insulin-secreting cells within these scaffolds, regardless of the organ source, improved insulin expression and efficiency in response to high-glucose stimuli *in vitro*. Furthermore, the implantation of recellularized scaffolds with insulin-producing cells at the subcutaneous site was effective in decreasing blood glucose (<15 mM) after 10 days, suggesting successful engraftment sustained by vascularization (169,172). The use of a native scaffold allowed the exploitation of pre-existing vessel structures within the decellularized organs to achieve reconstruction of the vasculature side of the endocrine pancreas. In fact, among these studies, only a few reports have seeded HUVEC within the native decellularized organs vasculature structure, achieving a successful reconstruction of the endothelial barrier *in vitro* and obtaining an *ex vivo* vascularized organ (Figure 2) (159,170). More interestingly, the dynamic culture of bioengineered devices has been shown to support the reconstitution of vasculature and to ameliorate the insulin secretion efficiency and viability of insulin-producing cells compared to those cultured in standard conditions, suggesting a successful *ex vivo* engraftment of endocrine cellular components (159,170). Our group used a decellularized rat lung left lobe to recreate a vascularized islet organ (VIO). HUVEC were seeded through pulmonary artery and vein, and the vasculature of the native lung was successfully recreated. Through the trachea, rodent islets were co-seeded with an additional amount of HUVEC, allowing them to reach the native decellularized alveolar structure, where they were retained. At this site, rodent islets receive metabolic support owing to the dense capillary network surrounding the alveoli, which are already vascularized (159). The relevant aspect was appreciated when the VIO platform was implanted in an immunocompromised diabetic murine model. In fact, its function was detected in almost 80% of recipient mice 5 days after subcutaneous implantation, demonstrating the importance of restoring vascularization *in vitro*. This allows a rapid vascular connection *in vivo*, shortening the hypoxic phase and limiting the loss of insulin-producing cells (159). These results demonstrate the tremendous impact of the ECM-shaped native-like architecture to favor pre-vascularization and engraftment *ex vivo*, which accelerates anastomosis and endocrine function *in vivo*. In this scenario, both endocrine pancreatic components and vascularizing elements were functionally and structurally intertwined owing to the coupled effect derived from the tailored ECM composition and microarchitecture of the decellularized organ.

To date, the use of native ECM to fabricate hydrogel scaffolds for tissue engineering is rapidly expanding because of the ease of decellularization. Upon decellularization, native ECM can be enzymatically digested to obtain smaller peptides that are useful for thermal-triggered hydrogels (dECM) (176–182). Hydrogels derived from porcine pancreas dECM have a beneficial role towards encapsulated rat islets, which secrete higher amounts of insulin than those encapsulated in alginate- or collagen-based hydrogels (183,184). Moreover, a recent study compared the impact of dECM derived from different porcine tissues (bladder, lung, and pancreas) on human and rodent islets. *In-situ* islet encapsulation within 3D-ECM hydrogels derived from the bladder and pancreas improves functional stability over standard culture conditions and enhances the retention of islet-resident EC (185). However, the resulting hydrogels gradually lost some of the native components, had poor mechanical properties, and were subjected to rapid degradation *in vivo*, without sustaining vascularization, leading to graft loss. Therefore, they are coupled with bio-inert materials showing poor degradability and higher stiffness, resulting in more suitable mechanical properties (112,186–188). Alginate capsules were generated to encapsulate insulin-producing cells dispersed within the dECM derived from human adipose tissue or porcine pancreatic tissue. Chemical modification of alginate with poly-L-lysine has also been proposed to increase the tolerance of the capsule by the immune system. This platform supported cell viability and differentiation and significantly improved insulin delivery, whereas *in vivo*, dECM-encapsulated cells were shown to be non-immunogenic and to significantly improve glycemic control in a diabetic preclinical model (188–191).

Hydrogels based on dECM showed good potential in terms of manipulation and production but were strongly limited by the loss of structure and microarchitecture. Hence, the control and reproducibility of spatial cell distribution are lost, as well as the ability to achieve total functional integration between endocrine and vascular compartments. Therefore, among the decellularized platforms, whole-organ engineering remains advantageous, as the native structure and composition might be completely exploited. Indeed, the results obtained from whole decellularized organs owing to their specific features have emphasized their promising capability in efficiently transplanting endocrine cells by recreating the native endocrine pancreatic niche. Furthermore, the flexibility of such systems might allow 1) their integration with valid alternative sources to human islets, and 2) exploitation of local immune-protection strategies for creating an immune-privileged endocrine site. With these important advances, bioengineered endocrine pancreas based on whole-organ decellularized scaffolds has the potential for clinical translation. Animal origin concerns might be overcome by using transgenic animal sources, limiting the xeno-reaction, and standardizing the procedures to obtain endotoxin-free scaffolds according to good manufacturing practices. Indeed, the use of animal tissue-derived ECM products has already been approved by the FDA and is commercially available for orthopedic surgery, and cardiovascular and skin repair (182).

### Reshaping the Architecture: 3D-Bioprinting Strategies

Lessons learned from whole-organ bioengineering highlighted that to recreate a microenvironment that is able to sustain the viability and function of both endocrine pancreatic and vascular compartments, the bioengineered scaffold should be provided with 1) a vascularized network and 2) a determined ECM composition with a hierarchical organization and microarchitecture (22). The use of ECM-derived components along with a wise design of bioengineered systems might be a good alternative strategy to match and functionally integrate these two features into unique bioengineered scaffolds, allowing the amelioration of both graft vascularization and endocrine viability and activity (22,112). 3D bio-printing technologies can be suitable for achieving tailored bioengineering devices for β-cell replacement, offering the following opportunities: 1) tuning the 3D spatial deposition of different cell types simultaneously, 2) encapsulation of cellular components within different hydrogel preparations, and 3) customizing the scaffold architecture according to the requested function (22,98,192) (Figure 2).

The 3D-printers available for this purpose differ with respect to the deposition methods (Table 1). Inkjet bioprinters are based on piezoelectric or thermal-driven mechanisms, allowing the deposition of a few microliters of a polymeric solution (193). They are poorly used because of the low cell density achievable in the structure compared to the physiological condition, as a high cell number may obstruct the nozzle. Additionally, the polymeric solution suitable for this system should not have a high viscosity, and thus the resultant structures are characterized by weak mechanical properties (194–196). Extrusion-based bioprinters consist of one or more nozzles that dispense the polymeric solution, namely bioinks, through pneumatic systems (air pressure or mechanical pistons) (193,197). These types of instruments are widely used in this research field as they are able to distribute an appropriate cell density in three-dimensional space, providing optimal structural integrity. With this system, the polymeric solution might have a wide range of viscosities, and the bioinks could be loaded with bioactive compounds, different cell types, cell aggregates, organoids, and tissue fragments (25,194,198). Additionally, it has a low printing speed and low spatial resolution, and, according to the nozzle diameter used, the pressure could affect cell viability (98). Finally, light-based printing strategies, such as stereolithography, exploit lasers to induce polymerization and deposition with high-resolution photo-crosslinkable polymers. Light-based printers accept a limited range of bioinks. Additionally, it considers encapsulating a finite number of cells resistant to the presence of a photoinitiator, which potentially exposes them to cell damage due to the generation of heat during polymerization (22,199). Among these setups, extrusion-based 3D printers are mostly used because of their flexibility, good biocompatibility, and minimal risk of damage to cellular components.

**TABLE 1:** Summary of the 3D bioprinting strategies and their possible advantages in β-cell replacement field.

3D printing strategies	Technical characteristics	Benefits for β-cell replacement
Inkjet-based bioprinting	Release of few microliters of hydrogel solution based on thermal or piezoelectric mechanisms	No published works exploiting this technique
	Use of low-viscous polymeric solutions	
	Low cells density	
Extrusion-based bioprinting	Extrusion of hydrogel solution through air pressure or mechanical pistons	Use of different type of cells
	Adjustable cells density	Possibility to provide a fine microenvironment composition and 3D structure
	Use of polymeric solutions with different viscosity	Spatial deposition for recreating pro-vascularizing structures
	Adjustable 3D spatial distribution	
Light-based printing	Deposition of polymers exploiting photo-initiators	Possibility to provide 3D pro-vascularizing structures
	Photo-crosslinkable polymers	
	Low cells density	
	Risk of cells damage	

3D-bioprinting is still in an exploration phase in β-cell replacement; therefore, research is focused on recreating not the whole pancreas, but the fundamental unit of the endocrine pancreatic tissue: insulin-producing and vasculature components supported by an ECM-based bio-mimicking scaffold. 3D-bioprinted scaffolds based on alginate/gelatin bioink encapsulating human islets showed improved islet viability *in vitro* (200)*.* However, insulin secretion analysis was conditioned by the high viscosity and reduced porosity of the hydrogel, which hindered glucose and insulin diffusion (200). To favor vessel formation, PCL was 3D-bioprinted in a porous ring scaffold, which was superficially modified with VEGF-binding heparin with a high degree of functionalization, while the human islet-encapsulating alginate solution was positioned in the ring hollows. The high surface-to-volume ratio provided by the specific porous structure, along with the slow release of VEGF, augmented the vascularization capability of the system *in vivo*. However, this study did not show the efficacy of the technology in sustaining endocrine function *in vivo* (104). These previous studies were limited in their ability to investigate and reconstitute the endocrine compartment and vasculature reconstruction without considering the functional and structural integration that exists in physiological conditions. Following this idea, another report used coaxial extruders, which allowed the fabrication of 3D-bioprinted alginate/methacrylate-gelatin (GelMA) strands with a core-shell structure, encapsulating EC and murine islets in the shell and core, respectively, obtaining uniform distribution of both cellular components in the bioengineered compartments (201,202). This system supported the islet viability, but the small pore size made it difficult to analyze insulin secretion (202). Given the key features of dECM in sustaining endocrine pancreatic components, recent studies have focused on the development of dECM-based bioinks for 3D-bioprinting. Porcine native pancreatic ECM (pdECM) embedded with insulin-producing cells and HUVEC was 3D-bioprinted, supporting the viability and function of β-cells and the pro-vascularizing ability of HUVEC *in vivo*, inducing optimal insulin secretion efficiency. This report validated the efficacy of pdECM as a source of bioink, demonstrating the possibility of recapitulating tissue-specific conditions in 3D constructs (183). Another study developed alginate/pdECM and alginate/fibrin bioinks to encapsulate porcine islets and HUVEC with MSCs. Alginate/pdECM hydrogel composition has been shown to sustain the viability and insulin secretion activity of porcine islets, whereas alginate/fibrin hydrogel supported the viability of HUVEC, inducing them to acquire sprouting morphology. Moreover, scaffolds with three different configurations were successfully fabricated, indicating that the complexity of the 3D-printed scaffold could be easily increased (203).

Despite the great advantage of precisely controlling cell deposition, it is worth emphasizing that cell organization changes over time through self-assembly mechanisms. Thus, there is a need to understand the underlying physiological processes behind in order to exploit them for the effective development and maturation of bio-mimicking structures, finalized to graft survival, integration, and function. Evidence from decellularized organs have highlighted the advantages of pre-vascularizing scaffolds in terms of insulin-producing cell viability and rapid *in vivo* revascularization and engraftment. 3D-bioprinted constructs designed for β-cell replacement should follow this strategy by creating channel or tubular architectures. Several bio-fabrication protocols have proposed different approaches for this purpose: use of coaxial nozzles to obtain hollow tubular strands; introduction of sacrificial polymers in extrusion-based bioprinting, such as Pluronic F127 or gelatin, which can be removed by changing the temperature, pH, or through enzymatic degradation, leaving hollow structures; and fabrication of perfusable light-based printed structures, which can be embedded in the 3D printed scaffolds (22,25,204–207). All these structures can be *in vitro* re-endothelialized to recreate a functional vasculature using a medium flow connected to a perfusable system, allowing a dynamic culture. This may promote cell infiltration and prompt revascularization *in vivo* (22). However, systems with this complexity have not been fabricated for β-cell replacement so far, not only because of the technological limitations of the 3D-bioprinting field, but also because of the biological issues concerning the sensitivity of insulin-producing cells and the intricacy of recreating the physiological mechanisms. To date, although investigation aimed at developing a bioengineered artificial endocrine pancreas through this type of technology is an attractive solution in the field of β-cell replacement, the advantageous use of a bioengineered decellularized whole organ for recreating the endocrine pancreatic niche remains a concrete and clinically relevant strategy for the treatment of T1D (Table 2).

**TABLE 2:** Summary of the bioengineering strategies aimed to improve the β-cell replacement.

Bioengineering strategies	Pros	Cons
Vascularizing the transplantation site	Increase the vascularization exploiting foreign body response	Delay of graft vascularization
	Release of proangiogenic factors	Passive and disorganized vessels formation
	Endocrine cells encapsulation grants the substitution of device upon exhaustion	Encapsulation hinders the ingrowth vessel formation
	Encapsulation grants also immune-protection	
Redrawing the endocrine cellular composition	Introduction of other cellular components for achieving biomimetic mechanisms to	Delay of graft vascularization
	• Increase vascularization	Disorganized vessels formation
	• Grant immune-protection	Scarce insights about the real immune-protection
	• Increase the viability and/or function of the endocrine cells
	Making insulin producing components homogenous in size to
	• Increase their viability and/or function	
	• Facilitate clinical procedures	
Reshaping the microarchitecture	Introduction of ECM components to provide the endocrine cells with suitable microstructures	Batch-to-batch differences
	Evidences on viability and function increasing thanks to ECM proteins	Need of standardized protocols
	Biomimetic cell-cell and cell-ECM interactions	*For 3D printing strategies*: lack of fidelity in recapitulating physiological structure and composition
	*Ex-vivo* pre-vascularization
	*In vivo* rapid graft vascularization	

## Insulin-Producing Cells: Finding an Alternative Source to Donor’s Islets

The identification of alternative and unlimited insulin-producing cell sources compatible with human implantation might fix donor organ shortage and broaden the clinical application of the treatment to a larger cohort of patients (208). To this aim, several solutions have been investigated developing differentiation protocols to β-cells derived from PSCs, as pluripotent embryonic stem cells (ESCs) or inducing-pluripotent stem cells (iPSCs) or evaluating xenogeneic sources.

### PSCs as a Source for β-Cell Replacement in T1D (ESC and IPSC)

ESCs and iPSCs are PSCs that are able to develop all three germinal layers of the embryo and therefore can differentiate into all cell types and tissues of the body (209). They can be guided to a specific cell fate by exposure to a defined combination of physical, chemical, and biological stimuli that can activate and/or inhibit specific signaling pathways that mimic human development. However, their high pluripotent hallmark represents a double-edged sword, as it is difficult to efficiently control their differentiation towards a specific cell fate. ESCs are isolated from the inner cell mass of blastocysts during embryonal development (210). Despite these advantages, there are some issues concerning their clinical translation, such as ethical concerns regarding their origin and allogeneic features. In 2006, Yamanaka et al. introduced the concept of reprogramming terminally differentiated somatic cells to an induced-PSC, forcing the expression of four key transcription factors, specifically Oct 4, Klf4, Sox2, and C-myc (211). iPSCs show similar features to ESCs, with the same morphology and proliferative rate, similar telomerase activity, normal karyotype, and the same *in vivo* teratogenous potential (ability to give rise to a teratoma, a germ layer tumor containing several types of tissues). Contrary to ESC, they had fewer ethical concerns and no allogeneic-related issues as they could be isolated from the patient itself. These features highlight the great potential of iPSCs for being used in clinical applications.

In the last 15 years, several authors have proposed protocols to reproduce step-by-step human pancreatic development *in vitro* to generate functional β-like cells from both ESCs and iPSCs (212). D’Amour et al. defined the first protocol to produce *in vitro* definitive endoderm from human PSCs, while later Kroon et al. demonstrated that ESC-derived pancreatic progenitors could further differentiate into glucose-responsive insulin-secreting cells after implantation into immune-deficient mice (213,214). Since then, several efforts have been made to understand and define a protocol for generating functional SC-derived β-cells *in vitro* that can secrete insulin in response to glucose stimuli. Pagliuca et al. were the first to report a scalable protocol to generate high numbers of functional SC-derived β-cells from both ESC and iPSCs of non-diabetic patients, with an average efficiency of 33% for β-like cells (215). Rezania et al. demonstrated the *in vivo* reversal of diabetes after the transplantation of SC-derived insulin-secreting cells. Although these insulin-secreting cells are similar to mature β-cells in terms of marker expression and insulin secretion, the differentiation protocol could not obtain cells fully equivalent to mature β-cells (216). In addition, the yield efficiency is still too low for clinical applications. In 2016, Millman et al. reported a scalable differentiation protocol to generate syngeneic β-cells from T1D of patients with iPSCs (217). Some years later, the same group demonstrated that differentiation towards β-like cell fate is guided by small molecules and growth factors and by cell-biomaterial interaction, which changes the cell cytoskeleton configuration and affects cell differentiation (218). Cells sense the microenvironment through integrin proteins that interact with the ECM, altering and/or promoting specific cellular processes. Thus, exploiting biomaterials to mimic ECM features, such as composition, stiffness, and geometry, might further improve differentiation protocols. After publication of these milestone differentiation protocols, several others came out with slight modifications, enhancing the quality of β-like cells generated *in vitro* and obtaining higher percentages of mono hormonal and insulin-expressing β-like cells (219–221). To achieve a successful clinical translation of PSC, there are several important challenges to be faced: 1) the lack of knowledge about the mechanisms that fully control cell differentiation towards all endocrine cell types, and adjustment of the ratio between β and non-β cells in the cluster to mimic the complexity and heterogeneity of human islet function, and 2) poor efficient strategies to protect PSC from immune rejection. Several clinical trials using ESCs and iPSCs have proposed different strategies to overcome these limitations. Viacyte investigated hESC-pancreatic progenitor cells transplanted within different encapsulation devices, VC-01 and VC-02, used in NCT02239354 and NCT03163511 trials, respectively (92–94). The VC-02 trial showed that hESC-pancreatic progenitor cells were successfully tolerated without teratoma formation. Moreover, they acquire a mature β-cell phenotype, as suggested by the analysis of explanted grafts (93,94,213). Finally, patients had increased fasting C-peptide levels and increased glucose-responsive C-peptide levels (93,94).

Vertex is another company that started a phase 1/2 clinical trial (NCT04786262), where the safety efficacy and tolerability of insulin-producing cells (VX-880) infused through the portal vein were evaluated. Recently, Vertex reported results from the first patient, who had a successful increase in fasting C-peptide and a decrease of exogenous insulin need by 91% over 90 days after implantation with half the target dose (222,223).

Finally, two other products, MailPan^®^ and Seraxis, use insulin-producing cells of different origins embedded in an immune-protective membrane. In conclusion, several efforts have been made to find an alternative source of islets, as indicated by numerous developed products and ongoing clinical trials (224,225).

### Xenogeneic Sources

The use of xenogeneic sources is another valid strategy for overcoming donor organ shortage. Previously, xenogeneic insulin from pigs has been adopted for human diabetes treatment for more than 60 years because of the amino acid similarity of porcine insulin to human insulin. The idea of using porcine islets as an alternative source of human islets was also derived from biological evidence. Porcine islets have the ability to respond to glucose stimuli within the same physiological range as human islets. Another advantage is the easy and reproducible isolation procedure. In contrast to human islet isolation, procedures adopted for porcine sources allow the preparation of high-quality porcine islets with good predictability and without being compromised by comorbidities, brain death, and ischemia. Additionally, porcine islets might be potentially used for highly allosensitized patients who present circulating antibodies against human leukocyte antigens (HLA), limiting the donors’ pool of compatibility with those patients (226).

Initially, the clinical use of porcine sources encountered some relevant limitations, especially related to the risk of zoonosis and more specifically, to the risk of porcine endogenous retrovirus (PERV) transmission (227). This can be overcome by genetic modification of the donor pigs. In a recent study, Yang et al. demonstrated the production of pigs with genetically inactivated PERVs using a combination of CRISPR-Cas9 and transposon technologies (228). Therefore, the use of animal sources coupled with advanced gene editing and cloning strategies has provided the opportunity to obtain genetically modified endocrine pancreatic sources, which can potentially cancel these concerns and improve their function. In this scenario, the low risk-benefit ratio of exploiting porcine islets as an alternative source to human islets makes them a promising option for the treatment of T1D (226).

Two fundamental aspects need to be considered when choosing the optimal porcine islet source: age and strain. Adult pigs can supply mature and large islets with the potential to efficiently secrete insulin within a few minutes or hours after transplantation, and the number of islets isolated from a sole adult pig might be sufficient for T1D patients (229). However, the disadvantages are principally related to the high costs of pig housing for an extended period before pancreas excision, and considering the need for endocrine sources owing to the wide diffusion of T1D disease, the costs can further increase. Moreover, islets from adult pigs have difficulties in isolation procedures and are fragile during culture (230). In contrast, neonatal islet-like cell clusters (NICCs) and fetal porcine islet-like cell clusters (FICCs) are easy and less expensive to isolate, as they have a relatively low cost of herd housing. In addition, isolation from fetal or neonatal porcine sources ensures procedures with a low contamination risk because of the ease of isolation in pathogen-free facilities. NICCs and FICCs do not present totally differentiated cells; therefore, they are prone to proliferation (231,232). In 1996, Korbutt et al. reported a simple, inexpensive, and reproducible method for isolating a large number of NCCIs (233). NCCIs consist of differentiated endocrine pancreatic cells and precursor cells, which showed *in vitro* and in preclinical studies to have the potential for proliferation and differentiation (232). Although NCCIs implantation in mice required at least 6–8 weeks to correct diabetes (233), when implanted in allogeneic pigs (234) or non-human primates (235), they demonstrated reversal of diabetes symptoms within 2–3 weeks. Therefore, they have the potential to increase endocrine cluster volume and, once matured, functionality after transplantation (236). In addition, they are more resistant to hypoxia, hyperglycemia, and pro-inflammatory cytokines than adult pigs (233,237). Elliot et al. performed a clinical trial of NCCIs transplants (NCT00940173). To date, 14 non-immunosuppressed patients with T1D have been treated with alginate-encapsulated NCCIs to alleviate and avoid the onset of hypoglycemic events (238). Separately from the metabolic improvement, none of the recipients showed signs of porcine viral infection, thus demonstrating the safety of the procedure. Nevertheless, increasing insulin production by NCCIs through genetic modification of genes involved in insulin granule exocytosis may be beneficial for their function. In particular, enhancing the response to glucose- and calcium-dependent depolarization *via* adenoviral transfer-mediated transgenic methods has been shown to increase the insulin stored within the granules and its secretion. This improved islet secretory function *in vitro*, bringing it closer to that of human islets and making them more efficient in controlling host glycemia in both preclinical and clinical trials, without the need to transplant a high number of islets (239,240). Another difference affecting the properties of old and young islets is related to ECM expression. In particular, islets from older pigs are isolated with higher ECM content than those from younger pigs (241). This may be reflected in the function of islets (242). Indeed, as for human islets, porcine islets are positively affected by ECM interaction, promoting islet cells survival, proliferation and efficient insulin secretion (243). More specifically, ECM proteins have been shown to be involved in modulating the differentiation of immature cells to mature cells (244). ECM content can also change depending on the pig breed. For example, German Landrace pigs have higher ECM protein expression and deposition in islet capsules than Deutsches Edelschwein pigs, facilitating isolation and permitting islets to be healthier for transplantation (245).

To date, the main disadvantages of all porcine endocrine cell clusters are their function-onset delay after transplantation and the high expression of oligosaccharide moieties, which trigger stronger cell and humoral-mediated immune rejections than allogeneic immune responses, rapidly leading to total xenograft rejection (246). Among the oligosaccharide groups, the most abundant are Galα1–3Galβ1–4GlcNAc-R (α-Gal), which is physiologically lower in adult pig islets (246,247) and is synthesized by α-1,3- galactosyltransferase (GGTA1), N-acetylneuraminic acid (Neu5Gc) synthesized by cytidine monophosphate-N-acetylneuraminic acid hydroxylase (CMAH), and an Sd(a)-like glycan made by β-1,4-N-acetyl-galactosaminyl transferase 2 (B4GALNT2) (248). Genetic engineering methodologies may facilitate the xenogeneic source compatibility with human. To abolish these carbohydrate antigens from porcine islets, pigs with knockout (KO) mutants of GGTA1, CMAH, and B4GALNT2, or a combination of these were generated. GGTA1-KO/CMAH- KO pigs did not show alterations in islet architecture or function. After transplantation of islets from these pigs into CMAH-deficient mice, no antibodies against Neu5Gc were detected (249). In addition, deletion of all three oligosaccharide antigens leads to considerably reduced human antibody binding to pig cells *in vitro* (250). In addition to delete xenogeneic genes, there is also the possibility to induce the expression of human genes, like CD55 and CD59 in α-Gal-deficient pig islets, which led to significantly high compatibility to the innate and adaptive immune system in humans. This strategy efficiently attenuates IBMIR after intraportal transplantation into immunosuppressed non-diabetic baboons *in vivo* (251).

## Immunosuppressive Strategies

The success of β-cell replacement is hampered by the poor engraftment capability of the graft in the peri-transplant phase and by the immunological reactions against the graft upon implantation. After intrahepatic transplantation, islets are exposed to the following: 1) IBMIR and innate immune reactions in the peri-transplant phase, 2) allogeneic immune recognition, and 3) recurrent autoimmune responses due to pre-existing adaptive immune memory (17). Thus, T1D patients receive life-long immunosuppressive treatment to prevent immune rejection (15,252,253). However, these treatments, although specific for the depletion of CD8 T-cells, are not able to completely target CD4 memory T-cells, accounting for autoimmunity recurrence (254–256). Additionally, chronic administration of immunosuppressive drugs results in severe systemic drawbacks and organ failure. In addition, some immunosuppressive drugs, such as tacrolimus and sirolimus, are toxic to β-cells (15). In this scenario, bioengineering approaches are not exclusively aimed at reshaping the endocrine pancreatic niche, but also at designing innovative strategies to overcome the immunological bottleneck.

Owing to the limitations of immunosuppressant strategies, the possibility of reconstituting the endocrine pancreatic niche by assembling the building blocks—insulin-producing cells, vasculature structure, and ECM-based microarchitecture—might be prone to the introduction of components able to locally immune-preserve the graft or modulate the host immune response, granting a long-term function (15). Bioengineering of an immune-protected vascularized endocrine device is challenging. It should not hamper the generation of vascular connections with the host, while it should promote both endocrine and vascular viability and function. This can be achieved by hiding the graft through encapsulation strategies or release of anti-inflammatory molecules, or by introducing components physiologically involved in immune-regulating mechanisms, making immune-stealth the endocrine pancreas device. Several strategies have been exploited for this purpose including the use of semipermeable membranes to physically immune-isolate the graft, chemical modification of the scaffold with anti-inflammatory or immune-modulating molecules, and the use of gene-edited cells expressing immune-modulating proteins.

### Immuno-Hiding the Endocrine Pancreatic Graft

The dimension of insulin-producing cells allows their encapsulation within biomaterial-based structures, which is useful for masking immunogenic antigens on cell surfaces and avoiding direct recognition by the host immune system (257–259). Currently, encapsulation with semi-permeable polymeric membranes is clinically investigated with the aim of hiding the bioengineered endocrine pancreas and blocking host immune cell infiltration and immunoglobulin and cytokine penetration, as well as allowing the diffusion of glucose, oxygen, and hormones (15,92–94). PTFE (92–94,225,260), alginate (122,261–265), agarose (266–268) and polyethylene glycol (PEG) (259,269–271) polymers have been used because of their anti-fouling and immune-inert features, ease of manipulation, and inclination towards chemical modifications. Various polymers and geometric configurations provided protection to islets in rodent models but failed to show benefits in large-scale animal models and clinical trials, especially because of FBR, which hampered the vascularization of the endocrine graft (122,264,272–274). Carefully tailoring the physicochemical and biological features of biomaterial-based encapsulation devices might reduce FBR mechanisms. The pore size plays an important role and therefore needs to be finely designed by adjusting the polymer molecular weight, concentration, composition, crosslinking degree, and porogen properties (273,275,276). Gradual degradation of the encapsulated biomaterials might provide a minor host immune response, allowing a more biomimetic graft integration process (277–279). Hence, innate immune cells and antigen presenting cells (APC) may switch towards a more tolerogenic phenotype, reducing the activation of adaptive immune response and potentially positively affecting long-term endocrine graft viability and function (280,281). Therefore, scalable engineering projects, comprehensive screening of FBR-inducing materials in preclinical models, and careful transplant site selection are required to strengthen translational effectiveness (122,272).

As of now, alternative bioengineered platforms aimed at immuno-hiding insulin-producing cells after implantation are designed either to integrate immune-instructive materials or to introduce immune-modulating cells or to deliver immune-modulating compounds for interfering with the locally inflamed microenvironment, reducing immunosuppressant side effects (282,283). Immunosuppressive molecules, such as mTOR and calcineurin inhibitors or mycophenolate mofetil (MMF), which are routinely systemically administered after clinical islet transplantation, can be locally delivered by the bioengineered devices for immuno-modulating the host response, decreasing their side effects (284–286). For instance, alginate-based beads modified with a “clickable” chemical group, complementary to another chemical moiety attached to rapamycin, have been implanted in the subcutaneous space of immunocompromised mice. Once consumed, it is refilled with complementary rapamycin, potentially providing continuous local immunosuppressive activity (284). However, some of these immunosuppressive drugs, such as fingolimod, which did not show adverse effects in preclinical studies upon systemic administration, may exhibit toxicity towards β-cells when locally delivered (287). Immune-modulating chemokines can be used for this purpose. CXCL12 linked to alginate scaffold-encapsulating islets has been shown to impair host T cell effector populations, granting graft long-term viability and function (288,289).

### Making Immune-Stealth the Endocrine Pancreatic Graft

Recently, the continuous understanding of immunological processes, such as immune tolerance, has opened the way for their potential exploitation in suppressing the host response after organ transplantation or triggering the host immune system against the tumor mass in cancer treatment (290,291). Immune tolerance involves a range of active processes that modulate or prevent potentially harmful immune responses and differ from immune ignorance, in which the immune system does not notice or recognize danger signals (292). Immune tolerance can be divided into two main categories: central and peripheral, with multiple layers of active regulation (293). Central tolerance refers to the mechanism by which immature T-cells are educated in the thymus. This selection induces apoptosis of T-cells with either too low affinity for HLA or too high reactivity to self-proteins expressed in the thymus. Finally, the selected T-cells can recognize peptides presented by HLA but do not respond to self-peptides (294). However, these central tolerance mechanisms are not impeccable, and self-reactive T-cells against islet autoantigens are frequently found in the circulation of healthy individuals, even if they do not manifest autoimmune disease (295). Therefore, the difference between healthy individuals and patients with autoimmune diseases must be researched in the role of these types of cells in peripheral tissues as well as in the efficacy of peripheral tolerance-regulating mechanisms (295). On the other hand, peripheral tolerance occurs in mature CD4 T and B cells, which are normally inhibited upon recognition of self-antigens (296). In addition, depending on the density of the antigen in peripheral tissues, immune cells may not respond to immunomodulatory co-stimulation, resulting in their inactivation (297). The players involved in peripheral tolerance induction are immune-modulating molecules such as cytotoxic T-lymphocyte antigen 4 (CTLA-4), programmed death (PD)-1, PD-ligand 1 (PD-L1), and Fas-ligand (FasL), which can decrease immune cell activation and correct the immune response.

Within the context of bioengineered systems, the use of immune tolerance induction could be capitalized on making devices with immune-stealth properties. The combination of endocrine pancreatic cells with cells expressing these proteins, such as MSCs or hAEC, which are physiologically involved in immunomodulation, has been exploited in previous studies, suggesting that they can potentially prevent graft rejection (140–142,298). Additionally, the identification of these specific proteins has opened the way for a combinatorial approach by modifying the bioengineered platform to generate an immune stealth device. For instance, PD-L1 directly linked to the islet surface increased graft survival in 90% of recipients, while, when it was linked to alginate, only 58% of recipients showed long-term graft function (299,300). Despite these results, exploiting this strategy in combination with material-based devices might facilitate its clinical translation. FasL is a molecule that causes T-cell apoptosis when linked to the cell surface or ECM, while its soluble form is anti-apoptotic. FasL materials have been fabricated and have demonstrated a positive impact on long-term graft function (301,302).

## Conclusion

Endocrine cells are structures enclosed by a BM-ECM layer that separates them from exocrine tissue and is fed by independent vasculature formed by a dense network of capillaries. The specific organization of the three building blocks, including the vasculature, ECM-based architecture, and insulin-producing cells, is essential for the physiological function of the endocrine pancreatic niche. In recent years, understanding their importance has become crucial to ameliorate β-cell replacement strategy outcomes, especially in improving the engraftment efficiency of insulin-producing cells. Bioengineering the transplantation sites using inert biocompatible materials to increase vascularization and shorten the hypoxic phase has been the most investigated approach in current clinical trials. However, these studies were principally focused on immune-preserving endocrine grafts and secondarily on increasing vascularization. In fact, semi-permeable membranes have been shown to hinder host immune system penetration and impede the migration of vascular cells, delaying the re-establishment of functional vascularization. The consequent loss of the graft highlights the necessity to develop strategies to trigger prompt graft vascularization rather than to grant graft immune protection, at least during the first phase of implantation. The use of more biomimetic approaches, such as introducing proangiogenic molecules or cells, redrawing the endocrine cellular composition with the addition of accessory cells, has been shown to ameliorate the rate of vascularization and consequently the treatment outcomes in preclinical studies; however, they did not fully reproduce the endocrine pancreatic native niche complexity. The missing part in those studies was the consideration of the endocrine pancreatic micro-architectural features because they allow the structural and functional integration between the vasculature and the endocrine components, as demonstrated by evidence from the positive results with the dECM organ used for bioengineering the vascularized endocrine pancreas. The preserved vessel structures of native organs allowed vasculature *in vitro* reconstruction. Additionally, the ECM-based microarchitecture, along with its specific composition, promotes full intertwining between the endocrine system and vasculature, ensuring rapid engraftment and function onset *in vivo*. In this scenario, the method for bioengineering a vascularized endocrine pancreas is paved as it should integrate insulin-producing cells, pro-vascularizing elements, and ECM-based scaffolds mimicking the endocrine pancreatic native niche. The use of 3D-bioprinting technologies might help to condense the building blocks in a fine-tuned bioengineered vascularized endocrine platform, exploiting its ability to finely fabricate a scalable microstructure encapsulating different cell types simultaneously. However, nowadays, the use of dECM organs is more ready for a possible clinical translation, as demonstrated by other dECM-based devices already used in clinical practice.

Bioengineering a vascularized endocrine device may also take advantage of alternative sources to human islets, overcoming donor organ shortages. PSC and xenogeneic source are valid alternative that can be easily integrated in bioengineered devices. Although PSC have been used in clinical studies, differentiation protocols are still not completely optimized. On the other hand, xenogeneic sources are endocrine cellular elements naturally assembled and prone to accomplish endocrine function, and additionally, they are easy to isolate. Concerns are related to their human immune-compatibility, which are easily surmountable with *ad-hoc* gene-editing strategies, as recently reported by gene-edited xenogeneic kidney and heart transplantation in human. Finally, bioengineered vascularized endocrine pancreas platforms are suitable for integrating novel immune-preserving strategies to ensure local immune modulation. Delivering immune-modulating molecules from the device or introducing immune-modulating cells are feasible strategies owing to the flexibility of tissue engineering fabrication methodologies.
